# Central Hypothyroidism with Low TSH Compared to Normal TSH Is Associated with More Advanced Pituitary Disease and Less Favorable Metabolic Profile

**DOI:** 10.3390/metabo15020125

**Published:** 2025-02-13

**Authors:** Aleksandra E. Matusiak, Jan Stępniak, Krzysztof C. Lewandowski, Andrzej Lewiński, Małgorzata Karbownik-Lewińska

**Affiliations:** 1 Department of Endocrinology and Metabolic Diseases, Medical University of Łódź, Rzgowska 281/289, 93-338 Łódź, Poland; aleksandra.matusiak@umed.lodz.pl (A.E.M.); jan.stepniak@umed.lodz.pl (J.S.); pl (K.C.L.); 2 Polish Mother’s Memorial Hospital—Research Institute, Rzgowska 281/289, 93-338 Łódź, Poland; 3 Faculty of Medicine, Mazovian University in Płock, Dąbrowskiego 2, 09-402 Płock, Poland

**Keywords:** hypothyroidism, thyrotropin, pituitary diseases, hypopituitarism, hyperlipidemias, thyroid hormones

## Abstract

**Background:** Central hypothyroidism is characterized by either decreased TSH or, more commonly, normal TSH. This study aims to check whether this biochemical difference related to the severity of the pituitary disease, metabolic processes and general well-being. **Methods:** A retrospective analysis was performed on 108 inpatients with hypopituitarism, aged 18–80, hospitalized (1 January 2020, through 31 December 2022) in the Department of Endocrinology and Metabolic Diseases, Medical University of Lodz, Poland. Hypopituitary patients with central hypothyroidism (*n* = 90) were divided into two subgroups: patients with TSH below normal ranges (low TSH; *n* = 52) and patients with TSH in reference ranges (normal TSH; *n* = 38). **Results:** Among patients with central hypothyroidism, surgical treatment due to pituitary disease was performed more commonly in those with low TSH than in those with normal TSH (65 vs. 42%, *p* = 0.010). Expectedly, five pituitary deficiencies were diagnosed more commonly in patients with low TSH than in those with normal TSH (46 vs. 13%, *p* = 0.001). In a regression analysis, the ACTH concentration was the only independent determinant negatively associated with low TSH (also after limiting the analysis to non-treated patients). Regarding lipid profile, decreased HDL cholesterol occurred more commonly in patients with low TSH vs. normal TSH (44% vs. 23%; *p* = 0.033), which was also observed after the limitation to non-treated patients (47% vs. 21%; *p* = 0.013). **Conclusions:** Low TSH in patients with central hypothyroidism is associated with more advanced pituitary disease and less favorable metabolic profile.

## 1. Introduction

Although an isolated failure of thyrotrope cell function can be observed [1], central hypothyroidism is usually associated with multihormonal hypopituitarism (combined pituitary hormone deficiencies), and the hypothyroid manifestations may be masked by the concomitant other pituitary defects [2].

The prevalence of central hypothyroidism has been estimated to range from 1:16,000 to approximately 1:100,000 [3]. It is reported to affect individuals of all ages and both sexes equally [4].

Central hypothyroidism can be either congenital or acquired. The manifestation of its congenital form usually begins in infancy, but has sometimes a delayed onset during childhood or adulthood. There are many causative genes for its isolated form or for multihormonal hypopituitarism, such as TSHβ, TRHR, TBL1X and IRS4 (for heritable isolated central hypothyroidism) and PROP1, HESX1, SOX3 (for multihormonal hypopituitarism) [5,6].

Approximately 50% of cases of acquired central hypothyroidism are caused by pituitary macroadenomas, whereas craniopharyngiomas are the most common extrasellar cause, especially in younger patients [7]. The frequency of central hypothyroidism due to nonfunctioning pituitary adenomas may reach 43% preoperatively and 57% postoperatively. Central hypothyroidism occurs in up to 65% of patients irradiated for brain tumors and up to half of patients irradiated for nasopharyngeal or paranasal sinus tumors. Central hypothyroidism due to traumatic brain injuries or stroke may increase in prevalence as more patients survive these events [8].

It is well known that secondary endocrine gland (the adrenal gland, the thyroid gland, and the gonads) deficiencies due to pituitary disease are usually associated with milder clinical symptoms compared to those caused by primary deficiencies. Accordingly, the clinical manifestations of central hypothyroidism are usually milder than those observed in patients with primary hypothyroidism [6].

Central hypothyroidism is characterized by a defect of thyroid hormone production due to insufficient stimulation by thyrotropin (TSH) of a normal thyroid gland. Whereas low TSH is expected, most patients with central hypothyroidism have TSH in reference ranges [6,9]. It is unknown whether this biochemical condition has any significance regarding metabolic processes.

It has been documented in several studies that hypothyroidism contributes substantially to the development of hyperlipidemia, because thyroid hormones modulate cholesterol production, transformation and its clearance. Therefore, the possible association of central hypothyroidism with abnormal lipid profile was also evaluated in the present study.

Taking into account the above observations our retrospective analysis aims to check whether the severity of the pituitary disease and/or routinely measured blood laboratory and anthropometric parameters in patients with central hypothyroidism differ between these with low TSH and those with normal TSH and whether these potential differences relate somewhat to the general well-being.

## 2. Materials and Methods

The procedures used in the study were approved by the Ethical Committee of the Medical University of Lodz, Poland (No. RNN/53/24/KE, 13 February 2024).

A retrospective analysis comprised the data of 108 consecutive inpatients with hypopituitarism (54 females, 50%), aged 18–80, hospitalized (2020–2022) in the Department of Endocrinology and Metabolic Diseases, Medical University of Lodz, Poland. Thus, the data of all patients hospitalized in the period of three years, in whom at least one pituitary hormone deficiency was diagnosed, were considered in the current study. The exclusion criteria were acute or chronic diseases; however it should be stressed that none of our hypopituitary patients had to be excluded from the analysis. We would like to point out that there was an overlapping of 10 patients who were retrospectively enrolled into the present study, and at the same time, they were prospectively enrolled to our previous study on pituitary diseases [10].

The patients were divided into two main groups:

- Patients with central hypothyroidism—90 out of 108 subjects (90/108; 83.3%)
- Patients without central hypothyroidism—18 out of 108 subjects (18/108; 16.6%); in these patients central hypothyroidism was excluded based on TSH, FT4 and FT3 levels being in normal ranges or based on normal results of stimulatory test with thyroliberin (TRH), the latter in a few cases. Therefore, these patients had either isolated growth hormone (GH) deficiency or GH deficiency together with gonadotropin deficiency.

Central hypothyroidism was diagnosed on the basis of [1,2]:

- Either low FT4 level together with low TSH
- Or low FT4 level together with TSH in normal ranges in the setting of pituitary disease; in a few cases, the diagnosis was confirmed by a stimulatory test with TRH.

In turn, hypopituitary patients with central hypothyroidism (*n* = 90) were further divided into two main groups:

- Patients with TSH below normal ranges (low TSH; *n* = 52)— 52 out of 90 subjects (52/90; 57.7%);
- Patients with TSH in reference ranges (normal TSH; *n* = 38)—38 out of 90 subjects (38/90; 42.2%)

Patients with central hypothyroidism (*n* = 90) [either with low TSH (*n* = 52) or with normal TSH (*n* = 38)] were further divided into two following subgroups:

- Patients with non-treated hypopituitarism (*n* = 49 + 33 = 82)—82 out of 108 subjects (82/108; 75.9%); these patients were newly diagnosed, thus without any replacement therapy.
- Patients with treated hypopituitarism (*n* = 3 + 5 = 8)—8 out of 108 subjects (8/108; 7.4%); these patients obtained appropriate replacement therapies regarding all pituitary hormone deficiencies for at least three months, and the hormonal compensation was confirmed by hormone target concentrations.

Among patients with low TSH, 51 out of 52 received L-thyroxine treatment (51/52; 98%). In the group of patients with central hypothyroidism and normal TSH, 27 out of 38 were on L-thyroxine replacement (27/38; 71%).

Most statistical analyses (when justified) were performed at 3 levels:

1. Hypopituitary patients (*n* = 108) with (*n* = 90) or without (*n* = 18) central hypothyroidism;
2. Patients with central hypothyroidism (*n* = 90) with low TSH (*n* = 52) or with normal TSH (*n* = 38);
3. Patients with non-treated hypopituitarism (*n* = 82).

Additionally, a few statistical evaluations were performed with the use of all hospitalization time points of patients with central hypothyroidism (*n* = 90 + 34 = 124; additional 34 hospitalization time points were recorded at the 2nd and—in few cases—at the 3rd time point for 30 patients).

**Figure 1:**
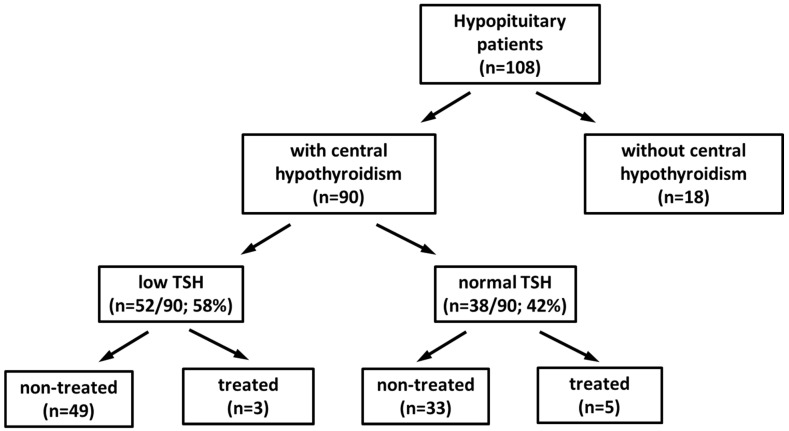
Study design diagram. Non-treated—newly diagnosed patients without any replacement therapy; treated—patients obtaining appropriate replacement therapies regarding all pituitary hormone deficiencies for at least three months (hormonal compensation was confirmed by hormone target concentrations).

The study design diagram is presented in Figure 1.

### 2.1. Blood Parameters

The concentrations of the following hormones and thyroid antibodies were measured in blood serum with the immunochemiluminescent method (Cobas e-601; Roche Diagnostics): thyroid-stimulating hormone (TSH), free triiodothyronine (FT3), free thyroxine (FT4), cortisol; adrenocorticotropic hormone (ACTH), prolactin (Prl), growth hormone (GH), parathyroid hormone (PTH), vitamin D and thyroglobulin antibody (TgAb), thyroid peroxidase antibody (TPOAb) and TSH receptor antibody (TSHRAb). The following other laboratory parameters were measured in blood serum with standard methods (Vitros 4600/Vitros 5.1; Johnson & Johnson, New Brunswick, NJ, USA): insulin-like growth factor I (IGF-I), total cholesterol (TChol), HDL cholesterol (HDLC), LDL cholesterol (LDLC), triglycerides (TGs), glucose, aspartate aminotransferase (ASPAT), alanine aminotransferase (ALAT), bilirubin, sodium (Na), potassium (K), chlorides (Cl), calcium (Ca) and C-reactive protein (CRP). Complete blood count was evaluated by the application of Sysmex XN-2000 Hematology System: red blood cells (RBC), hemoglobin (Hgb), white blood cells (WBC) hematocrit (HCT) platelets, neutrophils, lymphocytes, eosinophils, basophils and monocytes.

The following reference ranges for thyroid tests were accepted: TSH 0.27–4.2 mIU/L, FT4 0.93–1.7 ng/dL and FT3 2.0–4.4 pg/mL.

Body height and body mass were measured for the Body Mass Index (BMI) calculation.

### 2.2. Statistical Analyses

The data were statistically analyzed using Student’s unpaired *t*-test. The results are presented as means ± SEM. Univariate and multivariate logistic regression analyses were used to determine which continuous variable might have been associated with low/normal TSH or with non-treated/treated hypothyroidism. The two-sided ratio comparison test was used to evaluate the frequency of events. Statistical significance was determined at the level of *p* < 0.05.

## 3. Results

All continuous laboratory parameters in hypopituitary patients with central hypothyroidism and low TSH (*n* = 52) were compared to those obtained in hypopituitary patients with central hypothyroidism and normal TSH (*n* = 38) and also to those obtained in patients without central hypothyroidism (*n* = 18) (Table 1). Of importance, the groups did not differ regarding age and body mass. It is reasonable to list and, thereafter, to discuss the following statistically significant differences.

TSH concentration was obviously higher in patients without central hypothyroidism compared to patients with low TSH. In turn, the highest FT4 concentration was found in patients with low TSH, and FT3 concentration was higher in the low TSH versus normal TSH group. These apparently unexpected results regarding the highest FT3 and FT4 levels in patients with low TSH could be attributed to the fact that all patients but one with low TSH were on replacement therapy with L-thyroxine (Table 1).

Glucose concentration was higher in patients with normal TSH and in patients without central hypothyroidism compared to the group with low TSH. In turn, ACTH concentration was higher in patients with normal TSH and in patients without central hypothyroidism compared to the group with low TSH. Although the mean values of ACTH seemed to differ between patients with normal TSH and patients without central hypothyroidism (27.85 vs. 44.59), this difference did not reach the border of statistical significance. Regarding morning cortisol concentration, it was significantly higher in patients with normal TSH and in patients without central hypothyroidism compared to the low TSH group (Table 1).

**Figure 2:**
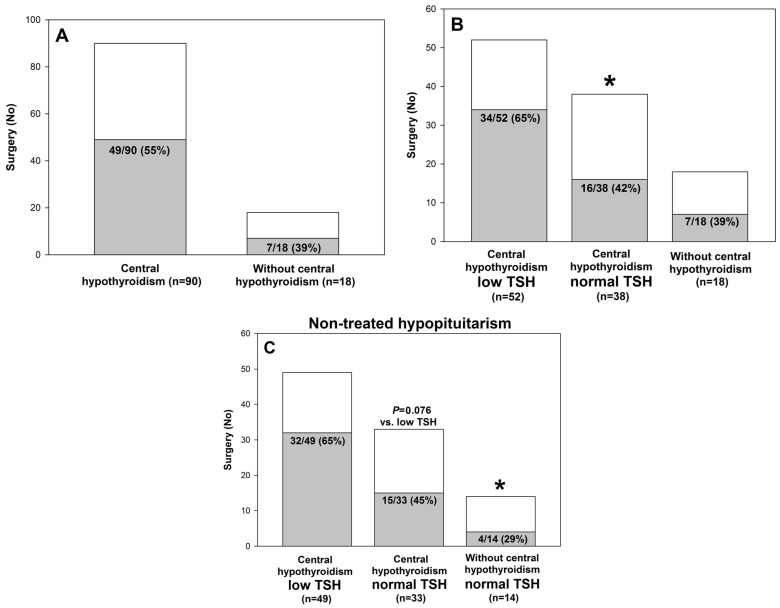
Number of hypopituitary patients in whom surgery was performed due to pituitary disease, presented in hypopituitary patients with central hypothyroidism (*n* = 90) vs. without central hypothyroidism (*n* = 18) (**A**), in patients with central hypothyroidism and low TSH (*n* = 52) vs. normal TSH (*n* = 38) and also vs. without central hypothyroidism (*n* = 18) (**B**) and in non-treated patients with central hypothyroidism and low TSH (*n* = 49) vs. normal TSH (*n* = 33) and also vs. non-treated patients without central hypothyroidism (*n* = 14) (**C**). The percentage of patients in whom surgery was performed is marked in gray. * *p* < 0.05 vs. low TSH.

We evaluated the number of patients in whom surgery was performed due to pituitary disease. Among hypopituitary patients with central hypothyroidism, surgical treatment due to pituitary disease was performed more commonly (*n* = 49/90; 55%) than in hypopituitary patients without central hypothyroidism (*n* = 7/18; 39%) (Figure 2A). When patients with central hypothyroidism were divided into low TSH and normal TSH subgroups, the number of pituitary surgeries was higher, as expected, in the former subgroup (*n* = 34/52; 52% vs. 16/38; 42%) (Figure 2B). The difference was even more pronounced in non-treated patients with central hypothyroidism, i.e., 31/49 (65%) vs. 14/33 (45%) for low TSH vs. normal TSH, respectively (Figure 2C).

Of importance is the finding that among hypopituitary patients with central hypothyroidism five pituitary deficiencies were diagnosed more commonly in those with low TSH than in those with normal TSH (*n* = 24/52; 46% vs. 5/38; 13%; *p* = 0.001) (Table 2A), and this difference was also seen when the statistical evaluation was performed among non-treated hypopituitary patients with central hypothyroidism (*n* = 24/49; 49% vs. 6/33; 18%; *p* = 0.005) (Table 2B).

Univariate/multivariate logistic regression analyses were performed to determine which continuous variables were associated with low TSH (Table 3). In the univariate analysis conducted in a group of hypopituitary patients with central hypothyroidism (*n* = 90) the following three determinants, glucose, ACTH and cortisol concentrations were negatively associated with low TSH, whereas—unexpectedly—erythrocyte level and hematocrit were positively associated with low TSH. In the multivariate logistic regression analysis, ACTH turned out to be the only independent determinant negatively associated with low TSH (Table 3A).

When the univariate regression analysis was performed in the group of non-treated hypopituitary patients with central hypothyroidism (*n* = 82), the same relationships were found as described above for the group of 90 patients. Additionally, hemoglobin and sodium level were positively associated with low TSH. In the multivariate regression analysis, the only determinant negatively associated with low TSH was, as in the previous analysis, ACTH concentration (Table 3B).

Another univariate logistic regression analysis was performed with the use of all hospitalization time points (*n* = 124). There were only three determinants negatively associated with low TSH, i.e., glucose, ACTH and cortisol concentrations. Once again, in the multivariate logistic regression analysis, the only determinant negatively associated with low TSH was ACTH concentration (Table 3C).

We evaluated the percentage of abnormal lipid profiles in particular subgroups among all hypopituitary patients (*n* = 108) (Table 4A) or among non-treated hypopituitary patients (*n* = 101) (Table 4B). The decreased HDL cholesterol was found more frequently in patients with low TSH comparing to normal TSH as well as comparing to patients without central hypothyroidism, and these differences were statistically significant when all hypopituitary patients were considered (Table 4A) and also when only non-treated hypopituitary patients were considered (Table 4B).

Univariate/multivariate logistic regression analyses were performed to determine which continuous variables were associated with inadequate hormone replacement therapies (non-treated hypopituitarism) (Table 5).

Among hypopituitary patients with central hypothyroidism (*n* = 90), only IGF-1 concentration was negatively associated with non-treated hypopituitarism. In multivariate regression analysis this parameter lost its statistical significance (Table 5A).

When all hospitalization time points (*n* = 124) were considered, two determinants, i.e. ACTH and IGF-1 concentrations, were negatively associated with non-treated hypopituitarism in the univariate regression analysis. In the multivariate logistic regression analysis the only determinant negatively associated with non-treated hypopituitarism was IGF-1 concentration (Table 5B).

When the percentage of abnormal lipid profile was evaluated among patients with central hypothyroidism (Table 6A) or in all hypopituitary patients (Table 6B), no statistically significant differences were recorded in a subgroup of non-treated vs. treated hypopituitary patients.

For additional statistical analyses, the low TSH (<0.27 IU/L) group was subdivided into patients with very low (<0.1 IU/L) and low (0.1–0.27 IU/L) TSH and the following parameters were compared between these subgroups: number of hypopituitary patients in whom surgery was performed, number of pituitary deficiencies, and percentage of HDLC < 40 mg/dL. Although no statistically significant differences were found it is worth mentioning that 5 pituitary deficiencies were diagnosed in 21/40 (52%) patients with very low TSH vs. 3/12 (25%) patients with low TSH (*p* = 0.105).

## 4. Discussion

We observed in the present study that among patients with central hypothyroidism there is an association of low TSH with: more common surgical treatment due to a pituitary disease, a higher number of pituitary axes involved (more pituitary deficiencies) and inadequate ACTH/cortisol secretion and abnormal lipid profile. Therefore, the following mechanisms of such phenomena should be considered.

It is expected that surgery was performed more commonly in patients with low TSH versus normal TSH, because the former group can have more advanced associated pituitary disease —at least in some cases—with larger tumors requiring to be removed surgically. The observations from the literature confirm that the risk and severity of post-surgical hypopituitarism depends on tumor size, its extension and also the experience of a neurosurgeon [11]. Concerning craniopharyngiomas, an example of large, typically slow-growing extrasellar tumors, surgical intervention is associated with hypopituitarism in most patients and central hypothyroidism has been reported in 78–95% of patients [12]. Although our patients were not treated with radiotherapy, it is worth mentioning that irradiation of the hypothalamic–pituitary region can cause hypopituitarism. Expectedly, the risk of developing central hypothyroidism is related to the effective dose given, which is equal to the degree of destruction of the hypothalamus or the pituitary gland [13].

In accordance with the above five pituitary deficiencies were recorded more commonly in patients with low TSH than in individuals with normal TSH, which can be explained by larger tumors as well as by more commonly performed pituitary surgery.

Again, in agreement with the above discussion, the association of low TSH with lower ACTH and lower cortisol concentrations results probably also from more advanced pituitary disease, i.e., a larger tumor and more frequently performed surgical treatment. Our observation has an important practical significance. Namely, patients with central hypothyroidism who have low TSH are at higher risk of secondary adrenal insufficiency (caused by ACTH deficiency). This is especially important in case of patients in whom it is difficult to unequivocally diagnose secondary adrenal insufficiency. Thus, low TSH strongly suggests the coexistence of adrenal insufficiency and, therefore, in patients who are not treated yet with hydrocortisone, careful monitoring should be recommended.

The relationship between TSH levels and the levels of other pituitary hormones, especially ACTH and GH, are complex and therefore they should be briefly discussed in relation to the result of the current study. As thyroid hormones (stimulated by TSH) and GH suppress adrenal axis and, on the other hand, cortisol suppresses TSH secretion [14], the parameters measured in our study could possibly be affected by these interactions. As our patients were either treated (obtaining all optimal replacement therapies) or non-treated (before starting replacement therapies), the above impact can be neglected. However, this aspect should be explored further in the course of gradual implementations of indicated replacement therapies.

Regarding lipid profile our findings were expected and are in agreement with what is commonly observed in hypopituitary patients. Of great importance is our previous observation showing that the increased total cholesterol was observed more frequently in patients with pituitary diseases, especially in those with hypopituitarism, compared to healthy individuals, and total cholesterol concentration was found to be the only independent factor associated with hypopituitarism [10]. Such relationship may result mostly from two pituitary deficiencies, namely GH deficiency [15,16] and TSH deficiency (causing central hypothyroidism) [17]. Thyroid hormones play an important role in the direct regulation of lipogenesis, fatty acid β-oxidation, cholesterol synthesis and the reverse cholesterol transport pathway. As a result, hypothyroidism can be associated with increased serum levels of cholesterol, triglycerides, low-density lipoprotein and apolipoprotein B [18]. Not only hypothyroidism but also isolated hypothyroxinemia negatively affects lipid metabolism resulting in abnormal lipid profile [19,20]. Interestingly, even short-term severe hypothyroidism caused by thyroid hormone withdrawal in post-thyroidectomy patients can lead to rapid significant and unfavorable changes in blood lipid levels [21]. In our previous study we confirmed that even high-normal TSH [22] is associated with certain unfavorable changes in lipid profile. Similar observations concerning atherogenic lipid profile apply to patients with secondary hypothyroidism. However, there are some differences in lipid profile constellation between primary and central hypothyroidism. In comparison with primary hypothyroidism, secondary hypothyroidism is characterized by a lower high-density lipoprotein cholesterol level [17] and by higher total triglyceride levels [17]. It is also worth mentioning that type IIb hyperlipidemia is the one that is most common in patients with central hypothyroidism, whereas type IIa hyperlipidemia is the most common lipid abnormality in patients with primary hypothyroidism [17].

Regarding our patients with central hypothyroidism, other pituitary hormone deficiencies such as ACTH/cortisol deficiency and GH/IGF-1 deficiency also contribute to a worse lipid profile, disease severity and overall quality of life (QoL). Regarding lipid abnormalities and GH/IGF-1 concentrations, making it more specific, GH deficiency can lead to increased levels of total cholesterol, low-density lipoprotein cholesterol and triglycerides, while high-density lipoprotein cholesterol is reduced, and there is an improvement in lipid profile after GH substitution [23].

Because lower ACTH, lower cortisol or also lower IGF-1 concentrations are probably results of a more advanced (more severe) pituitary disease, the overall quality of life of these patients is much more impaired than of patients with fewer axes involved. It is well documented that adult patients with GH deficiency, and at the same time with IGF-1 deficiency, have decreased QoL [24]. Regarding cortisol deficiency, it is also associated with impaired QoL [25]. Similar consequences can be expected in the case of ACTH deficiency causing secondary adrenal insufficiency. Interestingly, thyroid hormone replacement in central hypothyroidism is considered to be more difficult than in primary hypothyroidism, and regarding QoL, patients with central hypothyroidism scored worse in terms of depressive and emotional symptoms and impaired daily and social life than patients with primary hypothyroidism [26].

It should be stressed that documented differences between low and normal TSH regarding the number of pituitary deficiencies, surgery performed due to pituitary disease, abnormal lipid profile and inadequate function of the adrenal axis were independent of treated/non-treated hypopituitarism and, additionally, the results were confirmed when all hospitalization time points were considered. Such findings suggest that a more advanced pituitary disease and a less favorable metabolic profile are directly associated with lower TSH in patients with central hypothyroidism.

The differences between patients with low TSH and normal TSH discussed above are additionally strengthened by the fact that all patients but one with low TSH were on replacement therapy with L-thyroxine. Because—due to general recommendations—blood thyroid hormone levels should be kept in the upper normal range during replacement therapy, our patients with low TSH had the highest concentrations of circulating thyroid hormones. It should be stressed again that although patients with low TSH were properly replaced with thyroid hormones, they still had less favorable metabolic profiles.

It is strongly suggested that several TSH ranges (below lower normal range) should be considered in future studies comprising a much higher number of patients. Such a suggestion is supported by the higher (however not statistically significant) percentage of patients with five pituitary deficiencies found in our study in individuals with very low (<0.1 IU/L) vs. low (0.1–0.27 IU/L) TSH.

The question arises why a certain number of patients with central hypothyroidism have normal or even mildly elevated TSH with clearly decreased thyroid hormone concentrations. The answer to this question can be based partially on some facts presented in the literature, but also on our speculation. One should remember that surgery is likely to be a cause of low TSH (as well as a predictor). If thyrotrophs are preserved in patients with central hypothyroidism, TSH secreted by the pituitary gland can be devoid of full biological activity due to, for example, an altered glycosylation state. This qualitative defect in TSH secretion could explain why in a certain number of patients, the concentration of TSH is in normal ranges and at the same time, it could explain the lack of correlation between circulating levels of thyroid hormone and TSH concentrations [3,6]. What is more, TSH may reflect pituitary volume, assuming a lack of feedback from FT4. In turn, it can be speculated that in some patients with central hypothyroidism, TSH-producing cells still continue to work, they defend themselves against destruction and, consequently, they are able to respond more effectively to TRH stimulation. Therefore, in patients with normal TSH there can be still residual thyrotroph function, which–together with the physiological constitutive activity of the TSH receptor [9], results in the preserved, although decreased, function of the thyroid.

As mentioned in the Introduction section, to properly diagnose central hypothyroidism is still nowadays a challenge due to the lack of accurate clinical and biochemical parameters, especially when TSH remains within reference ranges or is above the normal range. According to the Endocrine Society Clinical Practice Guideline of Hormonal Replacement in Hypopituitarism in Adults it is recommended to measure serum FT4 and TSH to evaluate central hypothyroidism [2,8]. FT4 level below the laboratory reference range in conjunction with a low, normal, or mildly elevated TSH in the setting of pituitary disease usually confirms a central hypothyroidism diagnosis. At the same time dynamic TSH-secretion testing is not clearly recommended [2,8]. However, some authors recommend performing the stimulatory test with thyroliberin (TRH) in specific situations of suspected central hypothyroidism [27].

As already mentioned above, the timeline of hospitalization of our pa-tients overlaped significantly with the COVID-19 pandemic. This fact could theoretically affect pituitary function and consequently, our results regarding low TSH vs. normal TSH in patients with central hypothyroidism. Such an assumption is based on the numerous published reports on reversible hypophysitis and other transient endocrine disturbances following SARS-CoV-2 infection [28,29]. This raises the possibility that low TSH levels in some patients might not be directly related to the pituitary disease but rather reflect transient conditions triggered by viral infection or related inflammatory responses. Regarding the current study, none of our patients had COVID-19 infection documented either in the past or during hospitalization. It should be stressed that analyzing TSH values pre- and post-COVID-19 infection in future studies could help differentiate between persistent pituitary dysfunction and transient reversible changes.

The question arises regarding the significance of the current findings. Although clear differences were expected between hypopituitary patients with low TSH versus normal TSH regarding biochemical parameters, stage of the disease and general well-being, such results have not been published till now. Thus, the practical application may be as follows. Whereas both groups of patients, i.e., with low TSH and with normal TSH, should obtain the replacement therapy with levothyroxine, the dose of this medication should probably be higher in the former group, and these individuals with low TSH would probably have a lower chance for complete recovery. Regarding other replacement therapies, a higher number of them and higher doses of replacement medications are probably required in patients with low TSH. In hypopituitary patients but with an unclear extent of pituitary disease, low TSH argues in favor of other pituitary deficiencies, such as secondary adrenal deficiency, and this is an important aspect of the present study. Concerning biochemical/metabolic consequences of pituitary hormone deficiencies, they are probably more pronounced in patients with low TSH, which was documented in the current study by a worse lipid profile. Additionally, patients with low TSH were more frequently operated on due to their pituitary disease, and this fact does constitute a potential factor unfavorably affecting general well-being. Summarizing the significance of the current findings, patients with central hypothyroidism and low TSH are more unhealthy, and they require to be more carefully monitored for other pituitary deficiencies and, consequently, for systemic abnormalities.

It is also worth briefly discussing our observation that IGF-1 level was negatively associated with non-treated hypopituitarism. This relationship is in agreement with what was observed in hypopituitary adults in whom IGF-1 levels reflected hypopituitarism severity [30].

In the current study, we analyzed retrospectively the data of patients hospitalized in the Department of Endocrinology and Metabolic Diseases, Medical University of Lodz, Poland from January 2020 till December 2022, i.e., within the period of 3 years. The number of patients recorded i.e., 108 individual patients (or 124 hospitalizations), was unexpectedly low compared to previous years in our department ([10]; in that study patients were not enrolled consecutively but very selectively). Hypothetically, this apparent discrepancy could result from COVID-19 pandemic, which changed the profile of hospitalizations.

Our study has obviously some limitations. The first one is associated with a relatively low number of patients, which—although they were recorded within three years—probably resulted from pandemic issues. Another one is that GH deficiency was not diagnosed with the standard test, i.e., insulin tolerance test [8], and that most of our patients were not on replacement therapy with recombined human GH. This is due to the fact that the national program on recombined human GH treatment in adults has just recently been confirmed in Poland [31].

## 5. Conclusions

In conclusion, patients with central hypothyroidism and with low TSH have more advanced pituitary disease and less favorable metabolic profile compared to those with normal TSH. If low TSH is found in patients with central hypothyroidism, this may help to definitely diagnose other pituitary deficiencies and to predict less favorable course of hypopituitarism. Our findings suggest that individuals with low TSH in the course of central hypothyroidism require more careful observations for possible other pituitary deficiencies and consequently, for metabolic disturbances.

## Figures and Tables

**Table 1:** Clinical/laboratory parameters in hypopituitary patients with/without central hypothyroidism. Hypopituitary patients with central hypothyroidism were further divided into patients with TSH below reference ranges (low TSH; *n* = 52) and with TSH within reference ranges (normal TSH; *n* = 38). Comparisons between every two subgroups were performed by a Student’s unpaired t-test. Statistical significance was determined at the level of *p* < 0.05. Statistically significant differences are shaded and *p* levels in the second column (normal TSH) apply to comparison with “low TSH”, or—in case of the third column (without central hypothyroidism)—they apply to the comparison with “low TSH” and “normal TSH” in the order as written.

	Central Hypothyroidism		
	Low TSH (*n* = 52)	Normal TSH (*n* = 38)	Without Central Hypothyroidism (*n* = 18)
Age [years]	43.86 ± 2.42; *n* = 51	47.24 ± 3.01; *n* = 38; *p* = 0.380	51.17 ± 3.98; *n* = 18; *p* = 0.126, *p* = 0.450
Body mass [kg]	81.27 ± 3.49; *n* = 50	80.80 ± 3.48; *n* = 37; *p* = 0.927	88.56 ± 5.69; *n* = 12; *p* = 0.290, *p* = 0.233
Height [cm]	166.38 ± 1.62; *n* = 50	169.53 ± 1.85; *n* = 37; *p* = 0.204	173.65 ± 2.66; *n* = 17; *p* = 0.026, *p* = 0.215
BMI [kg/m^2^]	29.12 ± 1.12; *n* = 50	27.81 ± 1.01; *n* = 37; *p* = 0.406	29.29 ± 1.39; *n* = 17; *p* = 0.935, *p* = 0.405
TSH [mIU/L]	0.067 ± 0.01; *n* = 52	1.47 ± 0.212; *n* = 38; *p* < 0.001	1.60 ± 0.18; *n* = 18; *p* < 0.001, *p* = 0.694
FT3 [pg/mL]	2.86 ± 0.13; *n* = 50	2.03 ± 0.09; *n* = 38; *p* < 0.001	2.65 ± 0.08; *n* = 18; *p* = 0.345, *p* < 0.001
FT4 [ng/dL]	1.36 ± 0.05; *n* = 51	0.88 ± 0.049; *n* = 38; *p* < 0.001	1.09 ± 0.04; *n* = 17; *p* = 0.006, *p* = 0.011
TPOAb [IU/mL]	11.14 ± 0.91; *n* = 25	19.80 ± 4.57; *n* = 23; *p* = 0.059	8.38 ± 0.86; *n* = 8; *p* = 0.115, *p* = 0.156
TgAb [IU/mL]	15.65 ± 1.97; *n* = 24	36.72 ± 14.28; *n* = 23; *p* = 0.143	50.31 ± 36.89; *n* = 8; *p* = 0.107, *p* = 0.677
TSHRAb [IU/L]	0.867 ± 0.055; *n* = 23	0.865 ± 0.047; *n* = 21; *p* = 0.986	0.97 ± 0.11; *n* = 8; *p* = 0.369, *p* = 0.312
TChol [mg/dL]	194.6 ± 6.29; *n* = 52	199.3 ± 8.31; *n* = 38; *p* = 0.645	185.94 ± 9.40; *n* = 18; *p* = 0.474, *p* = 0.335
HDLC [mg/dL]	46.62 ± 2.46; *n* = 52	53.03 ± 3.49; *n* = 38; *p* = 0.126	53.61 ± 3.48; *n* = 18; *p* = 0.139, *p* = 0.917
LDLC [mg/dL]	116.15 ± 5.61; *n* = 52	114.34 ± 6.87; *n* = 38; *p* = 0.837	113.29 ± 8.92; *n* = 18; *p* = 0.793, *p* = 0.928
TGs [mg/dL]	160.65 ± 12.49; *n* = 52	140.60 ± 10.06; *n* = 38; *p* = 0.247	147.94 ± 21.31; *n* = 18; *p* = 0.613, *p* = 0.732
Glucose [mg/dL]	81.13 ± 1.22; *n* = 52	88.03 ± 3.04; *n* = 38; *p* = 0.023	88.17 ± 3.55; *n* = 18; *p* = 0.019, *p* = 0.978
ACTH [pg/mL]	17.95 ± 2.46; *n* = 37	27.85 ± 2.90; *n* = 33; *p* = 0.011	44.59 ± 13.07; *n* = 14; *p* = 0.004, *p* = 0.085
Cortisol [ug/dL]	5.58 ± 0.97; *n* = 52	8.79 ± 1.19; *n* = 37; *p* = 0.036	14.73 ± 1.73; *n* = 18; *p* < 0.001, *p* = 0.006
Prl [ng/mL]	23.34 ± 12.49; *n* = 52	41.50 ± 16.02; *n* = 37; *p* = 0.368	41.96 ± 25.29; *n* = 18; *p* = 0.475, *p* = 0.988
IGF-1 [ng/mL]	69.24 ± 10.06; *n* = 52	76.90 ± 10.99; *n* = 38; *p* = 0.612	97.24 ± 12.32; *n* = 17; *p* = 0.137, *p* = 0.260
RBC [10^12^/L]	4.47 ± 0.06; *n* = 52	4.21 ± 0.09; *n* = 38; *p* = 0.017	4.53 ± 0.12; *n* = 18; *p* = 0.676, *p* = 0.041
Hgb [g/dL]	13.08 ± 0.22; *n* = 52	12.49 ± 0.26; *n* = 38; *p* = 0.081	13.57 ± 0.45; *n* = 18; *p* = 0.296, *p* = 0.031
HCT [vol%]	38.44 ± 0.56; *n* = 52	36.39 ± 0.75; *n* = 38; *p* = 0.029	39.12 ± 1.38; *n* = 18; *p* = 0.588, *p* = 0.065
WBC [10^9^/L]	6.89 ± 0.23; *n* = 52	6.90 ± 0.35; *n* = 38; *p* = 0.987	6.93 ± 0.36; *n* = 18; *p* = 0.930, *p* = 0.955
Neutrophils [10^9^/L]	4.00 ± 0.69; *n* = 52	3.15 ± 0.17; *n* = 38; *p* = 0.307	3.56 ± 0.26; *n* = 18; *p* = 0.714, *p* = 0.190
Lymphocytes [10^9^/L]	2.76 ± 0.10; *n* = 52	2.98 ± 0.19; *n* = 38; *p* = 0.311	2.54 ± 0.24; *n* = 18; *p* = 0.318, *p* = 0.194
Eosinophils [10^9^/L]	0.223 ± 0.023; *n* = 52	1.20 ± 0.97; *n* = 38; *p* = 0.238	0.252 ± 0.031; *n* = 18; *p* = 0.509, *p* = 0.503
Basophils [10^9^/L]	0.047 ± 0.003; *n* = 52	0.059 ± 0.008; *n* = 38; *p* = 0.124	0.045 ± 0.005; *n* = 18; *p* = 0.718, *p* = 0.246
Monocytes [10^9^/L]	0.533 ± 0.02; *n* = 52	0.498 ± 0.03; *n* = 38; *p* = 0.308	0.651 ± 0.04; *n* = 18; *p* = 0.005, *p* = 0.003
ASPAT [U/L]	29.24 ± 1.67; *n* = 49	31.06 ± 1.34; *n* = 37; *p* = 0.420	29.29 ± 2.02; *n* = 18; *p* = 0.991, *p* = 0.457
ALAT [U/L]	27.26 ± 2.99; *n* = 49	24.18 ± 1.99; *n* = 38; *p* = 0.422	28.29 ± 4.16; *n* = 17; *p* = 0.856, *p* = 0.316
Bilirubin [mg/dL]	0.605 ± 0.03; *n* = 47	0.909 ± 0.29; *n* = 33; *p* = 0.219	0.733 ± 0.11; *n* = 18; *p* = 0.143, *p* = 0.664
Vit D [ng/mL]	30.14 ± 1.94; *n* = 48	28.49 ± 2.66; *n* = 37; *p* = 0.608	26.55 ± 2.37; *n* = 15; *p* = 0.337, *p* = 0.665
CRP [mg/dL]	0.86 ± 0.07; *n* = 40	0.633 ± 0.05; *n* = 27; *p* = 0.032	0.554 ± 0.07; *n* = 13; *p* = 0.034, *p* = 0.404
Na [mmol/L]	139.40 ± 0.28; *n* = 52	138.10 ± 0.63; *n* = 38; *p* = 0.042	139.67 ± 0.28; *n* = 18; *p* = 0.650, *p* = 0.119
K [mmol/L]	4.23 ± 0.03; *n* = 52	4.22 ± 0.07; *n* = 38; *p* = 0.846	4.44 ± 0.09; *n* = 18; *p* = 0.010, *p* = 0.077
PTH [pg/mL]	41.30 ± 2.78; *n* = 45	38.28 ± 3.10; *n* = 34; *p* = 0.472	37.56 ± 3.70; *n* = 14; *p* = 0.491, *p* = 0.894

**Table 2:** Number of pituitary deficiencies diagnosed in patients with central hypothyroidism (*n* = 90) (**A**) or in non-treated patients with central hypothyroidism (*n* = 82) (**B**) divided into those with low TSH (*n* = 52 or *n* = 49, respectively) and with normal TSH (*n* = 38 or *n* = 33, respectively). Statistical evaluation was performed by the two-sided ratio comparison test. Statistical significance was determined at the level of *p* < 0.05. Statistically significant differences are shaded.

**(A)**			
**Number of Pituitary** **Hormone Deficiencies**	**Central Hypothyroidism (*n* = 90)**		
	**Low TSH (*n* = 52)**		**Normal TSH (*n* = 38)**
5	*n* = 24 (46%)		*n* = 5 (13%), *p* = 0.001
4	*n* = 17 (32%)		*n* = 16 (42%), *p* = 0.332
3	*n* = 6 (12%)		*n* = 8 (21%), *p* = 0.251
2	*n* = 3 (6%)		*n* = 7 (18%), *p* = 0.076
1	*n* = 2 (4%)		*n* = 1 (3%), *p* = 0.801
**(B)**			
**Number of Pituitary** **Hormone Deficiencies**		**Central Hypothyroidism** **Non-Treated Patients (*n* = 82)**	
		**low TSH (*n* = 49)**	**normal TSH (*n* = 33)**
5		*n* = 24 (49%)	*n* = 6 (18%), *p* = 0.005
4		*n* = 16 (33%)	*n* = 14 (42%), *p* = 0.409
3		*n* = 6 (12%)	*n* = 7 (21%), *p* = 0.274
2		*n* = 3 (6%)	*n* = 6 (18%), *p* = 0.090
1		0	0

**Table 3:** Univariate and multivariate logistic regression analysis of low TSH determinants (continuous variables) performed in patients with central hypothyroidism (*n* = 90) (**A**) or in non-treated patients with central hypothyroidism (*n* = 82) (**B**). Additionally, regression analyses were performed with the use of all hospitalization time points, i.e., *n* = 90 + 34 = 124; additional 34 hospitalization time points were recorded at the 2nd and—in few cases—at the 3rd time point for 30 patients) (**C**). Only statistically significant determinants are presented in the tables. Statistical significance was determined at the level of *p* < 0.05. Statistically significant differences are shaded.

**(A)**						
**Variable**	**Patients with Central Hypothyroidism (*n* = 90)**					
	**Univariate Regression**			**Multivariate Regression**		
	**OR**	**95%Cl**	***p***	**OR**	**95%Cl**	***p***
Glucose	0.957	0.919–0.998	0.038	0.983	0.947–1.020	0.365
ACTH [pg/mL]	0.961	0.930–0.992	0.015	0.946	0.902–0.991	0.020
Cortisol	0.941	0.885–1.000	0.048	1.046	0.939–1.166	0.412
RBC [10^12^/L]	2.866	1.172–7.010	0.021	0.664	0.0234–18.848	0.810
HCT	1.120	1.009–1.245	0.034	1.222	0.820–1.819	0.324
**(B)**						
**Variable**	**Non-Treated Patients with Central Hypothyroidism (*n* = 82)**					
	**Univariate Regression**			**Multivariate Regression**		
	**OR**	**95%Cl**	***p***	**OR**	**95%Cl**	***p***
Glucose	0.952	0.911–0.995	0.029	0.969	0.921–1.020	0.232
ACTH [pg/mL]	0.953	0.918–0.988	0.009	0.936	0.887–0.989	0.018
Cortisol	0.924	0.866–0.987	0.018	1.021	0.904–1.153	0.741
RBC [10^12^/L]	3.396	1.291–8.931	0.013	0.297	0.0062–14.043	0.537
HGB	1.368	1.013–1.848	0.041	1.175	0.208–6.647	0.855
HCT	1.154	1.027–1.296	0.016	1.305	0.598–2.847	0.503
Na	1.204	1.008–1.439	0.041	1.242	0.994–1.550	0.056
**(C)**						
**Variable**	**All Samples (*n* = 124)**					
	**Univariate Regression**			**Multivariate Regression**		
	**OR**	**95%Cl**	***p***	**OR**	**95%Cl**	***p***
Glucose	0.967	0.937–0.998	0.035	0.973	0.937–1.011	0.163
ACTH [pg/mL]	0.954	0.926–0.982	0.002	0.964	0.929–1.000	0.048
Cortisol	0.896	0.845–0.951	<0.001	0.973	0.892–1.061	0.531

**Table 4:** Percentage of abnormal lipid profile in hypopituitary patients (*n* = 108) with central hypothyroidism (*n* = 90) (**A**) or non-treated central hypothyroidism (*n* = 87) (**B**) versus those without central hypothyroidism (*n* = 18, (**A**); *n* = 14, (**B**)). Patients with central hypothyroidism were further divided into those with low TSH (*n* = 52 or *n* = 49, respectively) and those with normal TSH (*n* = 38 or *n* = 38, respectively). Comparisons between every two subgroups were performed by the two-sided ratio comparison test. Statistical significance was determined at the level of *p* < 0.05. Statistically significant differences are shaded and the *p* levels in the second column (normal TSH) apply to comparison with “low TSH”, or—in case of the third column (without central hypothyroidism)—they apply to the comparison with “low TSH” and “normal TSH” in the order as written.

**(A)**			
	**Central Hypothyroidism (*n* = 90)**		
	**Low TSH** **(*n* = 52)**	**Normal TSH** **(*n* = 38)**	**Without Central Hypothyroidism** **(*n* = 18)**
TChol ≥ 200 mg/dL	*n* = 24 46%	*n* = 20 53%, *p* = 0.513	*n* = 7 39%; *p* = 0.607 vs. low TSH; *p* = 0.332 vs. normal TSH
HDLC < 40 mg/dL	*n* = 23 44%	*n* = 9 23%, *p* = 0.033	*n* = 3 17%; *p* = 0.045 vs. low TSH; *p* = 0.609 vs. normal TSH
LDLC > 100 mg/dL	*n* = 33 63%	*n* = 22 58%, *p* = 0.632	*n* = 12 67%; *p* = 0.761 vs. low TSH; *p* = 0.522 vs. normal TSH
TGs > 150 mg/dL	*n* = 21 40%	*n* = 14 37%, *p* = 0.774	*n* = 4 22%; *p* = 0.173 vs. low TSH; *p* = 0.267 vs. normal TSH
HDLC/TChol < 0.2	*n* = 6 11%	*n* = 3 6%, *p* = 0.413	*n* = 1 6%; *p* = 0.539 vs. low TSH; *p* = 1.000 vs. normal TSH
**(B)**			
	**Non-Treated (*n* = 101)**		
	**Central Hypothyroidism (*n* = 87)**		**Without Central Hypothyroidism** **(*n* = 14)**
	**Low TSH** **(*n* = 49)**	**Normal TSH** **(*n* = 33)**	
TChol ≥ 200 mg/dL	*n* = 23 47%	*n* = 19 50%, *p* = 0.782	*n* = 5 36%; *p* = 0.468 vs. low; *p* = 0.373 vs. normal
HDLC < 40 mg/dL	*n* = 23 47%	*n* = 8 21%, *p* = 0.013	*n* = 2 14%; *p* = 0.029 vs. low; *p* = 0.571 vs. normal
LDLC > 100 mg/dL	*n* = 31 63%	*n* = 19 50%, *p* = 0.227	*n* = 10 71%; *p* = 0.582 vs. low; *p* = 0.182 vs. normal
TGs > 150 mg/dL	*n* = 20 40%	*n* = 13 34%, *p* = 0.567	*n* = 3 21%; *p* = 0.195 vs. low; *p* = 0.370 vs. normal
HDLC/TChol < 0.2	*n* = 6 12%	*n* = 3 8%, *p* = 0.543	*n* = 0

**Table 5:** Univariate and multivariate logistic regression analysis of non-treated hypopituitarism determinants (continuous variables) performed in patients with central hypothyroidism (*n* = 90) (**A**). Additionally, regression analyses were performed with the use of all hospitalization time points, i.e., *n* = 90 + 34 = 124; additional 34 hospitalization time points were recorded at the 2nd and the 3rd time point for 30 patients) (**B**). Only statistically significant determinants, or when justified, are presented in the univariate analysis. Statistical significance was determined at the level of *p* < 0.05. Statistically significant differences are shaded.

**(A)**						
**Variable**	**Patients with Central Hypothyroidism (*n* = 90)**					
	**Univariate Regression**			**Multivariate Regression**		
	**OR**	**95%Cl**	***p***	**OR**	**95%Cl**	***p***
IGF-1	0.991	0.983–0.999	0.024	-	-	-
**(B)**						
**Variable**	**All Samples (*n* = 124)**					
	**Univariate Regression**			**Multivariate Regression**		
	**OR**	**95%Cl**	***p***	**OR**	**95%Cl**	***p***
ACTH [pg/mL]	0.960	0.928–0.994	0.021	0.964	0.928–1.001	0.055
IGF-1	0.987	0.979–0.994	<0.001	0.985	0.975–0.996	0.005

**Table 6:** Percentage of abnormal lipid profile in patients with central hypothyroidism (*n* = 90) (**A**) or in all patients (*n* = 108) (**B**) divided into those who were non-treated (*n* = 82 and *n* = 96, respectively) and treated (*n* = 8 and *n* = 12, respectively). Statistical evaluation was performed by the two-sided ratio comparison test. Statistical significance was determined at the level of *p* < 0.05. Statistically significant differences are shaded.

**(A)**		
	**Non-Treated** **(*n* = 82)**	**Treated** **(*n* = 8)**
TChol ≥ 200 mg/dL	*n* = 42 51%	*n* = 2 25%, *p* = 0.163
HDLC < 40 mg/dL	*n* = 31 38%	*n* = 1 12%, *p* = 0.146
LDLC > 100 mg/dL	*n* = 50 61%	*n* = 5 62%, *p* = 0.912
TGs > 150 mg/dL	*n* = 33 40%	*n* = 2 25%, *p* = 0.407
HDLC/TChol < 0.2	*n* = 9 11%	*n* = 0
**(B)**		
	**Non-Treated** **(*n* = 96)**	**Treated** **(*n* = 12)**
TChol ≥ 200 mg/dL	*n* = 47 49%	*n* = 4 33%, *p* = 0.297
HDLC < 40 mg/dL	*n* = 33 34%	*n* = 2 17%, *p* = 0.237
LDLC > 100 mg/dL	*n* = 60 62%	*n* = 7 58%, *p* = 0.788
TGs > 150 mg/dL	*n* = 36 37%	*n* = 3 25%, *p* = 0.415
HDLC/TChol < 0.2	*n* = 9 9%	*n* = 1 8%, *p* = 0.907

## Data Availability

The data presented in this study are available upon request from the corresponding author. The data are not publicly available as they contain information that could compromise the privacy of research participants.
